# Central nervous system disease and genital disease in harbor porpoises (*Phocoena phocoena*) are associated with different herpesviruses

**DOI:** 10.1186/s13567-016-0310-8

**Published:** 2016-02-09

**Authors:** Cornelis van Elk, Marco van de Bildt, Peter van Run, Anton de Jong, Sarah Getu, Georges Verjans, Albert Osterhaus, Thijs Kuiken

**Affiliations:** Dolfinarium Harderwijk, Strandboulevard Oost 1, 3841 AB Harderwijk, The Netherlands; Department of Viroscience, Erasmus Medical Center, Wytemaweg 80, 3015 CN Rotterdam, The Netherlands; Department of Pathology, Erasmus Medical Center, Wytemaweg 80, 3015 CN Rotterdam, The Netherlands; Research Center for Emerging Infections and Zoonoses, University of Veterinary Medicine, Bünteweg 2, 30559 Hannover, Germany

## Abstract

Herpesvirus infection causes disease of variable severity in many species, including cetaceans. However, little is known about herpesvirus infection in harbor porpoises (*Phocoena phocoena*), despite being widespread in temperate coastal waters of the Northern Hemisphere. Therefore, we examined harbor porpoises that stranded alive in the Netherlands, Belgium, and Germany between 2000 and 2014 for herpesvirus infection and associated disease. Porpoises that died or had to be euthanized were autopsied, and samples were collected for virological and pathological analyses. We found one known herpesvirus (*Phocoena phocoena* herpesvirus type 1, PPHV-1)—a gammaherpesvirus—and two novel herpesviruses (PPHV-2 and PPHV-3)—both alphaherpesviruses—in these porpoises. A genital plaque, in which PPHV-1 was detected, occurred in 1% (1/117) of porpoises. The plaque was characterized by epithelial hyperplasia and intranuclear inclusion bodies that contained herpesvirus-like particles, and that stained positive by a PPHV-1-specific in situ hybridization test. PPHV-2 occurred in the brain of 2% (1/74) of porpoises. This infection was associated with lymphocytic encephalitis, characterized by neuronal necrosis and intranuclear inclusion bodies containing herpesvirus-like particles. PPHV-3 had a prevalence of 5% (4/74) in brain tissue, 5% (2/43) in blowhole swabs, and 2% (1/43) in genital swabs, but was not associated with disease. Phylogenetically, PPHV-1 was identical to a previously reported herpesvirus from a harbor porpoise, PPHV-2 showed closest identity with two herpesviruses from dolphins, and PPHV-3 showed closest identity with a cervid herpesvirus. In conclusion, harbor porpoises may be infected with at least three different herpesviruses, one of which can cause clinically severe neurological disease.

## Introduction

Herpesvirus infections can cause disease of variable severity in many species, including cetaceans [1]. The most common small cetacean species in the North Sea is the harbor porpoise (*Phocoena phocoena*) [2, 3]. This population of harbor porpoises is still vulnerable, according to IUCN criteria [4]. Therefore, monitoring of and research into morbidity and mortality factors is important for the conservation of the population. Specifically, trends in both prevalence and severity of herpesvirus infection potentially may provide information on changes in immune status of the harbor porpoise population, as these are known to increase when immune competence decreases [5, 6].

Mammalian herpesviruses belong to the ancient virus family *Herpesviridae* of the order *Herpesvirales*, which is subdivided into *Alphaherpesvirinae*, *Betaherpesvirinae* and *Gammaherpesvirinae* [7]. During cospeciation for millions of years, herpesviruses have adapted to their hosts resulting in a large diversity of herpesviruses with a restricted host range. Most mammalian species have at least one and frequently multiple herpesviruses, if they are looked for. This includes cetaceans, in several species of which herpesvirus infections have been documented. Herpesvirus-associated lesions in cetaceans range from mucosal and/or cutaneous lesions [8–10] to encephalitis [11–13] and disseminated systemic disease [14]. Mucosal lesions have been associated with *Gammaherpesvirinae* [9], encephalitis and disseminated systemic disease have been associated with *Alphaherpesvirinae* [11, 12, 14], and cutaneous lesions have been associated with *Alphaherpesvirinae* and *Gammaherpesvirinae* [9, 10]. Despite the fact that the harbor porpoise is a relatively common cetacean species and many stranded individuals in several countries have been autopsied [15–17], the only reported herpesvirus infections are a case of encephalitis associated with herpesviral antigen expression in affected neurons [12], and several cases of dermatitis, in which a gammaherpesvirus, tentatively named Phocoenid herpesvirus-1, was detected by PCR [10].

As part of a long-term study of morbidity and mortality factors of live-stranded harbor porpoises in the Netherlands, we here describe two cases of herpesvirus infection: a gammaherpesvirus (tentatively named *Phocoena phocoena* herpesvirus 1, PPHV-1) infection associated with a genital mucosal plaque, and an alphaherpesvirus (tentatively named *Phocoena phocoena* herpesvirus 2, PPHV-2) infection associated with encephalitis. In addition, we document a distinct alphaherpesvirus (tentatively named *Phocoena phocoena* herpesvirus 3, PPHV-3) in brain tissue, pulmonary lymph node, blowhole and genital slit. The data suggest that PPHV-3 has a higher prevalence of infection in live-stranded harbor porpoises than PPHV-2, both in the brain (5% [4/74] vs 2% [1/74]) and genital slit (2% [1/43] vs 0% [0/43]), but is not associated with disease. PPHV-1 was only found in association with genital plaque, which had a prevalence of 1% (1/117) in live-stranded harbor porpoises. These results indicate that herpesvirus infections in harbor porpoises are more common than previously known and may be associated with severe, potentially fatal disease.

## Materials and methods

### Rescue and rehabilitation of live-stranded cetaceans

Since 1967, small cetaceans—mainly harbor porpoises—that strand alive along the Dutch, Belgian and German coasts have been rehabilitated at the Dolfinarium Harderwijk (Harderwijk, The Netherlands) and subsequently released into the wild. Since 2004, this activity has been organized in the form of an independent foundation, SOS Dolfijn, which operates at the same site. SOS Dolfijn has two 50 m^3^ pools with fresh water to which salt is added. In the first period of rehabilitation, animals are observed 24 h per day and standard parameters are recorded, including respiration rate, cramps, food intake and defecation. In addition, other potentially relevant observations are recorded, including swimming behavior and alertness. As an animal improves, the level of observation and care diminish to a minimum of 9 h per day.

Between 2009 and 2014, the genital slits, blowholes and oral cavities of rescued harbor porpoises were sampled upon admission by use of a dry cotton swab for virological diagnosis. Swabs were stored in virus transport medium [Hank’s balanced salt solution supplemented with 0.5% lactalbumin, 10% glycerol, 200 U/mL penicillin, 200 μg/mL streptomycin, 100 U/mL polymyxin B sulfate, 250 μg/mL gentamycin, and 50 U/mL nystatin (ICN Pharmaceuticals)].

Admission and rehabilitation of live-stranded harbor porpoises at SOS Dolfijn was authorized by the government of the Netherlands (application number FF/75/2012/036). Samples used in the present study were collected from harbor porpoises for diagnostic purposes by qualified personnel of the SOS Dolphin Foundation under veterinary supervision. SOS Dolfijn provided permission to the Department of Viroscience, Erasmus Medical Center to use the samples for the present study. No samples were collected from animals for research purposes.

### Autopsy and histology

In recent years, survival of rehabilitated harbor porpoises at SOS Dolfijn has been close to 50%. Since 2000, those harbor porpoises that die or have to be euthanized, based on poor prognosis, have been autopsied at the Department of Viroscience (Erasmus MC, Rotterdam, The Netherlands) as part of a long-term program to understand morbidity and mortality factors of the North Sea harbor porpoise population.

Autopsies were performed according to a standard protocol [18]. The following tissues were sampled for histology: adrenal gland, bronchus, cerebellum, cerebrum, colon, duodenum, esophagus, forestomach, fundic stomach, gonads, heart, jejunum, kidney, liver, lung, mesenteric lymph node, muscle, pancreas, pulmonary lymph node, pyloric stomach, skin, spleen, thymus, thyroid, trachea, tracheobronchial lymph node, and urinary bladder. Tissue samples were fixed in 10% neutral-buffered formalin, routinely processed, and embedded in paraffin. The 3-μm-thick sections were mounted on glass slides and stained with hematoxylin and eosin (HE) for light microscopy.

### Electron microscopy

Formalin-fixed, paraffin-embedded samples of brain tissue and genital mucosa were deparaffinized and embedded in epoxy resin. Thin sections were prepared, stained with 6% saturated uranyl acetate and lead citrate, and examined with a Philips Morgagni 268D electron microscope (F.E.I., Brno, Czech Republic).

### In-situ hybridization

For detection of gammaherpesvirus RNA, 5-μm-thick tissue sections were stained with a commercial in situ hybridization (ISH) technique as described previously [19]. The probe was designed by Advanced Cell Diagnostics (Hayward, CA, USA), based on the 1246 base pair (bp) herpesvirus-specific DNA polymerase fragment obtained from next-generation sequencing and subsequent Sanger sequencing of tissue samples from porpoise #6 (Table 1). For ISH, the RNAscope 2.0 FFPE Assay (Advanced Cell Diagnostics, Inc.) was used according to the instructions of the manufacturer. In short, sections were deparaffinized in xylene, dehydrated in ethanol and subsequently pretreated to allow access to target RNA. The probe was hybridized for 2 h at 40 °C, signal was amplified with six amplification steps and finally the signal was visualized with Fast Red. The section was counterstained with hematoxylin and mounted with Ecomount.

**Table 1:** **Clinical and pathological evidence of central nervous system disease and genital disease in live-stranded harbor porpoises in which herpesvirus was detected**

Porpoise number	Pathology number	Age category	Gender	CNS disease			Genital disease		Herpesvirus PCR-positive samples	Tentative virus type
				Nervous signs	Encephalitis	INIB^a^	Mucosal plaque	INIB		
1	PP040324	Juvenile	Male	No	Yes	No	No	No	Brain	PPHV-3
2	PP050825.2	Juvenile	Female	Yes	Yes	Yes	No	No	Brain	PPHV-2
3	PP051021	Juvenile	Male	No	Yes	No	No	No	Brain, pulmonary lymph node	PPHV-3
4	PP030320	Juvenile	Male	No	No	No	No	No	Brain	PPHV-3
5	PP060327.4	Juvenile	Male	Yes^b^	No	No	No	No	Brain, cornea	PPHV-3
6	PP050502	Adult	Female	No	Yes	No	Yes	Yes	Skeletal muscle, urinary bladder, kidney, skin lesion	PPHV-1
7	PP140627^c^	Neonate	Female	No	No	No	No	No	Blowhole swab, genital swab	PPHV-3
8	N.a.^d^	Juvenile	Male	No	N.a.	N.a.	No	N.a.	Blowhole swab	PPHV-3

^a^Intranuclear inclusion bodies.

^b^Forceful expiration, body tremor, cramp.

^c^This porpoise was swabbed on arrival at rehabilitation centre and subsequently autopsied.

^d^Not applicable.

### Herpesvirus polymerase chain reaction and sequencing

The following porpoise tissues were sampled for polymerase chain reaction (PCR) (Table 1): brain (combined samples of cerebrum and cerebellum), genital plaque (porpoise #6 only), kidney, mammary gland cyst (porpoise #6 only), liver, lung, skeletal muscle (porpoise #6 only) spleen and urinary bladder. Tissue samples were stored at −70 °C until use. Tissue samples were thawed and homogenized using a Fastprep24 tissue homogenizer (MP Biomedicals, Santa Ana, CA, USA). From genital swab samples, a 200 µL aliquot was taken. DNA was isolated from tissue homogenates and swab samples using the High Pure Viral nucleic Acid Kit (Roche, Almere, The Netherlands), following the protocol provided by the manufacturer. A nested herpesvirus PCR was performed as described previously [20]. In brief, two forward primers (HV-F1: 5′-GAYTTYGCNAGYYTNTAYCC-3′ and HV-F2: 5′-TCCTGGACAAGCAGARNYSGCNMTNAA-3′) and one reverse primer (HV-R1: 5′-GTCTTGCTCACCAGNTCNACNCCYTT-3′) directed to the polymerase gene were used in the first PCR. An aliquot of 2 µL from the first PCR reaction was used for a nested PCR with one forward primer (HV-F3: 59-TGTAACTCGGTGTAYGGNTTYACNGGNGT-39) and one reverse primer (HV-R2: 59-CACAGAGTCCGTRTCNCCRTANAT-39). Products of the PCR reactions were checked by electrophoresis on a 2% agarose gel for fragments of the correct size. Automated sequencing of PCR fragments was performed on an ABI 3130XL genetic analyzer with the Big Dye Terminator Cycle Sequencing Kit (Applied Biosystems, Foster City, California, USA), using the nested herpesvirus PCR primers. For identification of sequenced fragments, the BLAST option of the National Center for Biotechnology Information website was used.

### Phylogenetic analysis

Phylogenetic analysis was performed by creating an alignment using the ClustalW method in MEGA6 [21], based on a polymerase gene fragment of the detected herpesviruses. A maximum likelihood phylogram was constructed using the maximum likelihood method based on the Kimura 2-parameter model with 500 bootstrap replicates.

### Next-generation sequencing and detection of herpesvirus sequences

Tissue samples of three harbor porpoises (brain from porpoise #1 and #2, and skeletal muscle from porpoise #6) were processed for sequence-independent DNA virus screening as described previously [22, 23]. In brief, tissues samples were defrosted and homogenized using a Fastprep24 tissue homogenizer (MP Biomedicals) in Hank’s balanced salt solution supplemented with 0.5% lactalbumin, 10% glycerol, 200 U/mL penicillin, 200 μg/mL streptomycin, 100 U/mL polymyxin B sulfate, 250 μg/mL gentamycin and 50 U/mL nystatin (ICN Pharmaceuticals, Laval, Quebec, Canada) and centrifuged briefly. Supernatants from homogenates were filtered and the samples were treated with Omnicleave Endonuclease (Epicentre Biotechnologies, Madison, WI, USA). Subsequently, viral nucleic acids were extracted using the High Pure Viral Nucleic Acid kit (Roche) according to the instructions of the manufacturer. Random amplification was performed and amplicons were processed for next-generation sequencing with a 454 GS Junior instrument (Roche). Reads were trimmed and assembled using de-novo assembly in CLC Genomics Workbench 5.5.1 (CLC Bio, Aarhus, Denmark) and analyzed by nucleotide and translated nucleotide BLAST searches. Sequences were classified based on the taxonomic origin of the best-hit sequence using MEGAN software. E-values of e^−10^ were used as the cut-off value of significant virus hits for BLASTn and BLASTx.

### Sanger sequencing

Using the 454-sequencing reads obtained from porpoise #6, open reading frame 56 (ORF56)-specific primers were designed based on ORF56 Ovine herpesvirus 2 to obtain partially overlapping PCR amplicons using AmpliTaq Gold DNA polymerase (Roche). The overlapping PCR fragments were sequenced, resulting in a 1246 bp fragment of the ORF56 homologue of the herpesvirus from porpoise #6 (GenBank accession number KU200258).

### Papillomavirus PCR

A papillomavirus-specific PCR was performed on the clitoral plaque sample from porpoise #6 using primers MY11/MY09 [24] and GP5+/GP6+ [25]. This PCR had been successful in detecting DNA of two papillomaviruses from Burmeister’s porpoise (*Phocoena spinnipinnis*) [26].

### Virus culture

Ten percent homogenates of selected tissue samples that had been stored at −70 °C were inoculated on the following cell types: primary harbor porpoise kidney cells, Madin-Darby bovine kidney cells, Madin-Darby canine kidney cells, and Crandell feline kidney cells. Cultures were washed twice with Dulbecco’s modified Eagle’s medium supplemented with antibiotics and 10% fetal calf serum and incubated at 37 °C. Cultures were checked daily for cytopathic changes for a maximum of 10 days. At least three passages were made before cultures were considered negative. At the end of each passage, all cultures were tested for the presence of herpesviral DNA by PCR as outlined above.

## Results

### Alphaherpesvirus PPHV-2 infection associated with encephalitis

Porpoise #2 was a juvenile female harbor porpoise (body weight 19.4 kg, standard body length 110 cm at autopsy) found alive on 11 July 2005 at Middelskerk (Oostende, Belgium) (Table 1). During the first 5 days after admission at the rehabilitation centre, the porpoise had difficulty to coordinate the surfacing of the blowhole and inspiration, resulting in inadvertent inspiration of water and coughing. During the next 3 days, the porpoise showed exaggerated surfacing with the entire head out of the water, possibly to prevent the inspiration of water. Breathing frequency remained within normal reference ranges (<5/min). Shivering or tremors were observed during the entire 8 days in rehabilitation. The porpoise was euthanized on 19 July 2005 because of poor prognosis.

**Figure 1:**
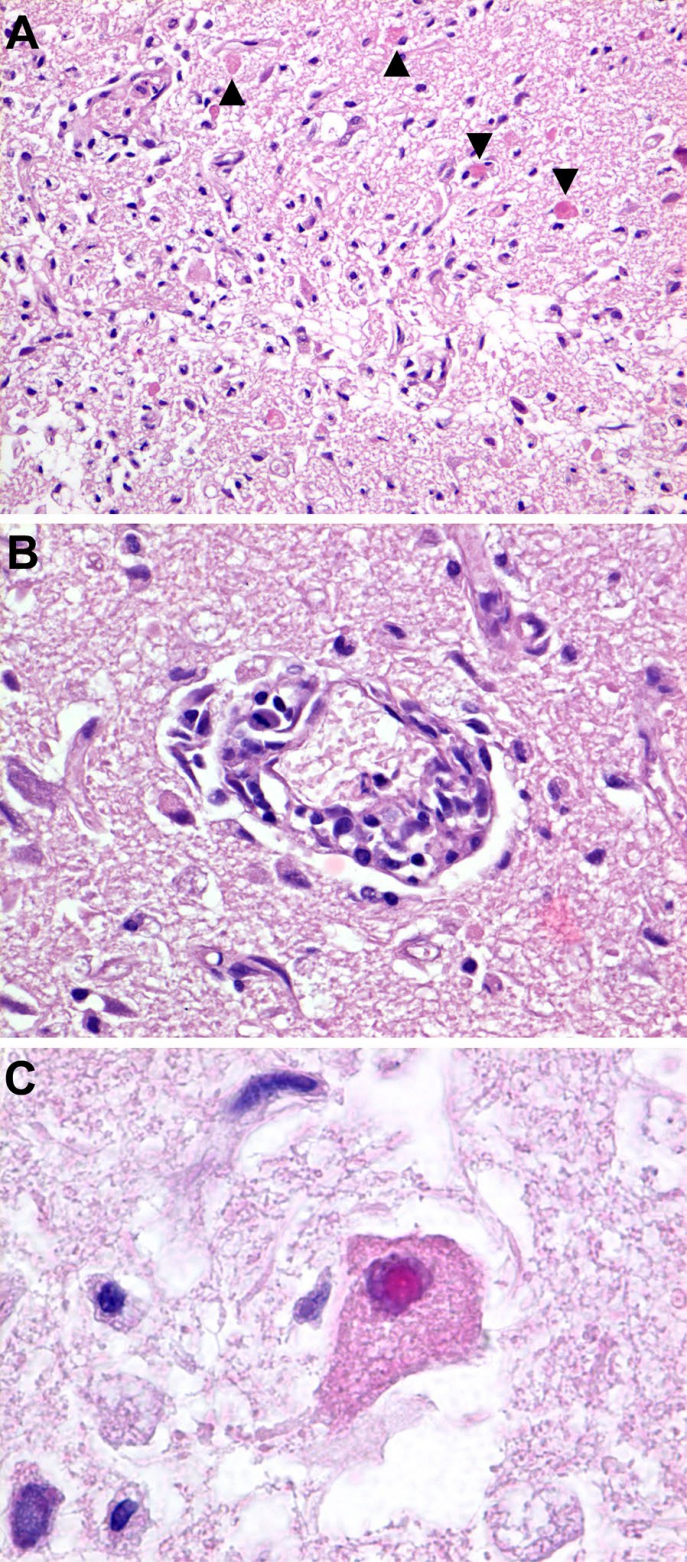
**Encephalitis associated with** ***Phocoena phocoena*** **herpesvirus type 2 infection in a harbor porpoise.** Cerebrum of harbor porpoise #2 infected with *Phocoena phocoena* herpesvirus type 2, an alphaherpesvirus. All tissue sections are stained with hematoxylin and eosin, and show mild freeze–thaw artifact. The encephalitis is characterized by an increased density of cells in the neuropil of the brain and the presence of dead neurons (arrowheads), which lack nuclei and have hypereosinophilic cytoplasm (**A**). In these areas, there are cuffs of mononuclear cells around blood vessels (**B**). Some of the affected neurons have eosinophilic intranuclear inclusion bodies, which are characteristic for herpesvirus infection (**C**).

**Figure 2:**
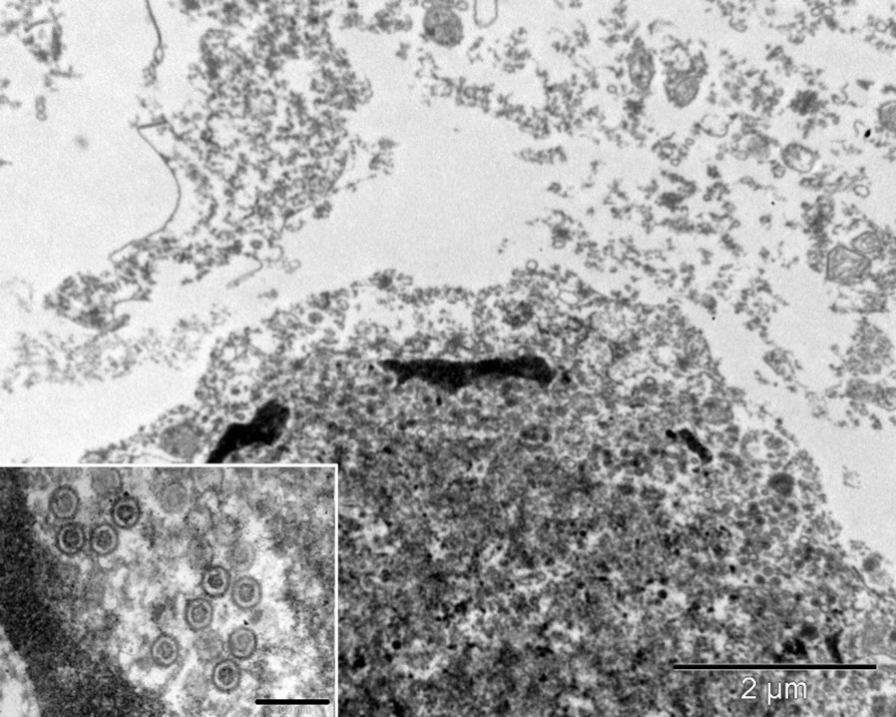
**Herpesvirus-like particles in neuron of a harbor porpoise with** ***Phocoena phocoena*** **herpesvirus type 2 infection.** Cerebrum of harbor porpoise #2 infected with *Phocoena phocoena* herpesvirus type 2, an alphaherpesvirus. Transmission electron micrographs of a neuron, stained with uranyl acetate and lead citrate. The nucleus of the neuron has electron-dense clumps of marginated chromatin and contains an intranuclear inclusion body. Within this inclusion body, there are round to hexagonal unenveloped viral nucleocapsids, some with an electron-dense core (inset). Bar of inset = 200 nm.

**Figure 3:**
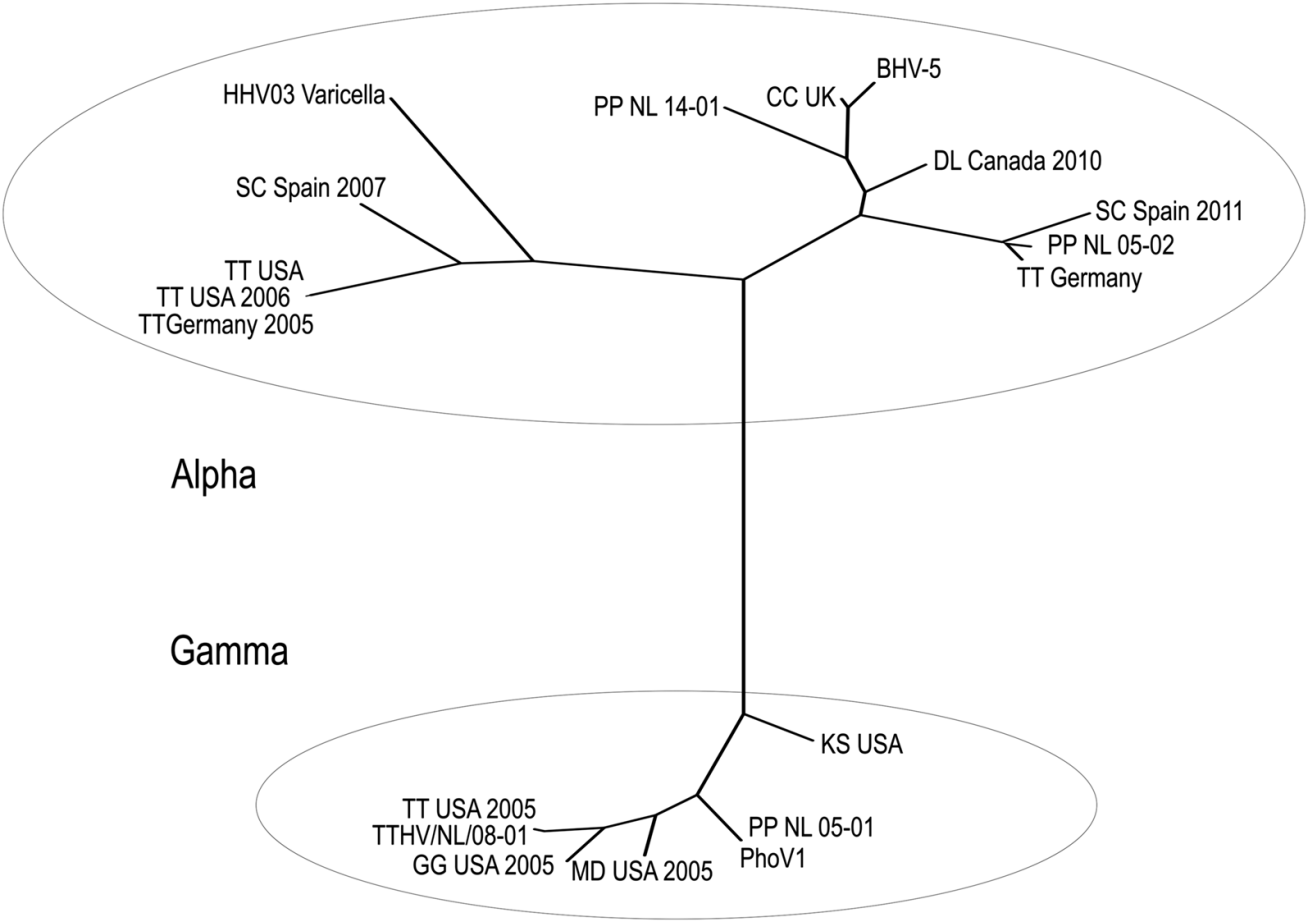
**Phylogenetic tree to compare harbor porpoise herpesviruses from this study with other marine mammal herpesviruses and other representative herpesviruses.** Maximum likelihood phylogram, based on the Kimura 2-parameter model, of DNA polymerase gene fragments for comparison of several porpoise herpesviruses with marine mammal herpesviruses and selected other herpesviruses. Bootstrapping was performed with 500 replicates using MEGA6 [21] GenBank accession numbers are given in parentheses: HHV03 Varicella: Human Herpesvirus—3 (X04370); SC Spain 2007: Striped dolphin Spain, 319Li_Sc, 2007 (GQ888673); TT Germany 2005: Bottlenose dolphin Germany, 2005 (DQ295064); TT USA 2006: Bottlenose dolphin USA, strain SPL1, 2006 (GQ429149); TT USA: Bottlenose dolphin USA (AF245443); CC UK: Cervid Herpesvirus, Banffshire 82, North American Elk (Cervus canadensis) (DQ328329); BHV-5: Bovine herpes virus—5 (AY261359); DL Canada 2010: Beluga Whale herpesvirus (Delphinapterus leucas), Canada 2010 (KJ789857); TT Germany: Bottlenose dolphin Germany (AY608707); SC Spain 2011: Striped dolphin Spain 2011 (KJ156331); TT USA 2005: Bottlenose dolphin USA 2005 (AY952779); TTHV/NL/08-01: Bottlenose dolphin Netherlands 2008 (GQ258353); GG USA 2005: Risso’s dolphin herpesvius, strain MML0514-V2172 (KJ406184); MD USA 2005: Blainville’s beaked whale K285, 2005 (AY949828); KS USA: dwarf sperm whale (Kogia simus), K265 (AY949830); PhoHV1: Phocoenid herpesvirus 1, UT775, 2012 (KT591613); PP NL 05-01: Porpoise #6, Netherlands, PP050502 (KT991635, this study); PP NL 05-02: Porpoise #2, Netherlands, PP050825.2 (KT991634, this study); PP NL 14-01: Porpoise #7, Netherlands PP140627 (PPSH198) (KT991633, this study).

The main pathological diagnosis was a lymphocytic encephalitis with neuronal necrosis and intranuclear inclusion bodies (INIB). No macroscopic changes were seen in the brain, but histologically in the cerebrum, there was a locally extensive area of increased density of nuclei in the gray matter. In this area, blood vessels had lymphocytic cuffs about three cells thick and randomly scattered neutrophils in the neuropil. In multiple neurons, the cytoplasm was eosinophilic and the nuclei had large amphophilic inclusion bodies and marginated chromatin (Figure 1). By electron microscopy, the nuclei of these neurons contained many round or hexagonal particles with a consistent diameter of approximately 90 nm (Figure 2). Most particles had a round, electron-dense core, whereas others were empty. Size, shape and location of these particles are consistent with herpesvirus nucleocapsids [27]. Other significant diagnoses in this porpoise were multifocal pyogranulomatous pneumonia associated with nematode infection (probably *Stenurus minor*), multifocal proliferative arteritis of the pulmonary arteries associated with nematode infection (probably *Pseudalius inflexus*), and diffuse pulmonary oedema associated with euthanasia. By PCR, herpesvirus DNA (PPHV-2) was detected in the brain sample of this porpoise. The viral DNA polymerase 201 bp amplicon showed strongest identity with an alphaherpesvirus of a bottlenose dolphin (*Tursiops truncatus*) from Germany (92%) and a striped dolphin (*Stenella coeruleoalba*) that died during the dolphin morbillivirus outbreak in Spain in 2011 (87%) (Figure 3). Other tissues from this porpoise (kidney, liver, lung, spleen and urinary bladder) tested negative for herpesvirus DNA by PCR. Virus culture from brain, lung, spleen, kidney and urinary bladder samples of this porpoise was attempted, but no virus was cultured.

Based on this case report, we retrospectively screened brain samples of live-stranded harbor porpoises that were autopsied between 2000 and 2014 (*n* = 74) for herpesvirus infection and encephalitis. The animals identified included 1 male abortion, nine male neonates, 25 male juveniles, seven male adults, six female neonates, 16 female juveniles and 11 female adults. Brain samples were tested for herpesvirus DNA by PCR and examined for histopathological changes by light microscopy. If brain tissues were found positive for herpesvirus DNA, other frozen tissues from the same porpoise were also tested. We found four additional porpoises (#1, #3 to #5) with herpesvirus DNA in the brain (Table 1). Two of these porpoises also had herpesvirus DNA in extra-neurological tissues: pulmonary lymph node and cornea (Table 1). The sequences of the PCR products from all four porpoises were identical to each other, but distinct from PPHV-2. The virus (PPHV-3) showed closest identity (86%) with cervid herpesvirus from a North American elk (*Cervus canadensis*) (Figure 3). Therefore, the overall prevalence of PPHV-2 and -3 infections in the brain of live-stranded porpoises that were autopsied was 1% (1/74) and 5% (4/74), respectively. Notably, PPHV-3 had a predeliction for juvenile male porpoises: 16% (4/25).

We found six additional porpoises with histopathological changes in the brain: three juvenile males and an adult female with inflammation of cerebrum, one adult male with inflammation of cerebrum, cerebellum and cervical spinal cord, and one juvenile female with inflammation of cerebellum and meninges. In general, these changes consisted of increased cell density in neuropil (gliosis), aggregation of macrophages around neurons (satellitosis), and aggregation of mononuclear cells around blood vessels (perivascular cuffing). Brain tissues of two (#1, #3) of these six porpoises also were positive for herpesvirus DNA (PPHV-3, Table 1). However, no neurons with INIB typical of herpesvirus infection were found in brain samples of any of these six porpoises. Consequently, it was not possible to attribute these histopathological changes to herpesvirus infection. In one porpoise, the juvenile female, the inflammation was associated with a mixed bacterial and fungal infection (data not shown). Therefore, the overall prevalence of encephalitis in live-stranded porpoises that were autopsied was 9% (7/74).

### Gammaherpesvirus PPHV-1 infection associated with genital plaque

Porpoise #6 was an adult female harbor porpoise (body weight 58.9 kg, standard body length 161 cm at autopsy) found alive on 24 November 2003 at Noordwijk aan Zee (The Netherlands). After long-term rehabilation at SOS Dolfijn the porpoise developed kidney failure. This was most evident from blood serum values: urea increased from 18 to 30 mmol/L, creatinine from <27 to 81 μmol/L and sodium from 158.5 to 196.4 mmol/L. The porpoise was euthanized on 2 May 2005 because of poor prognosis.

**Figure 4:**
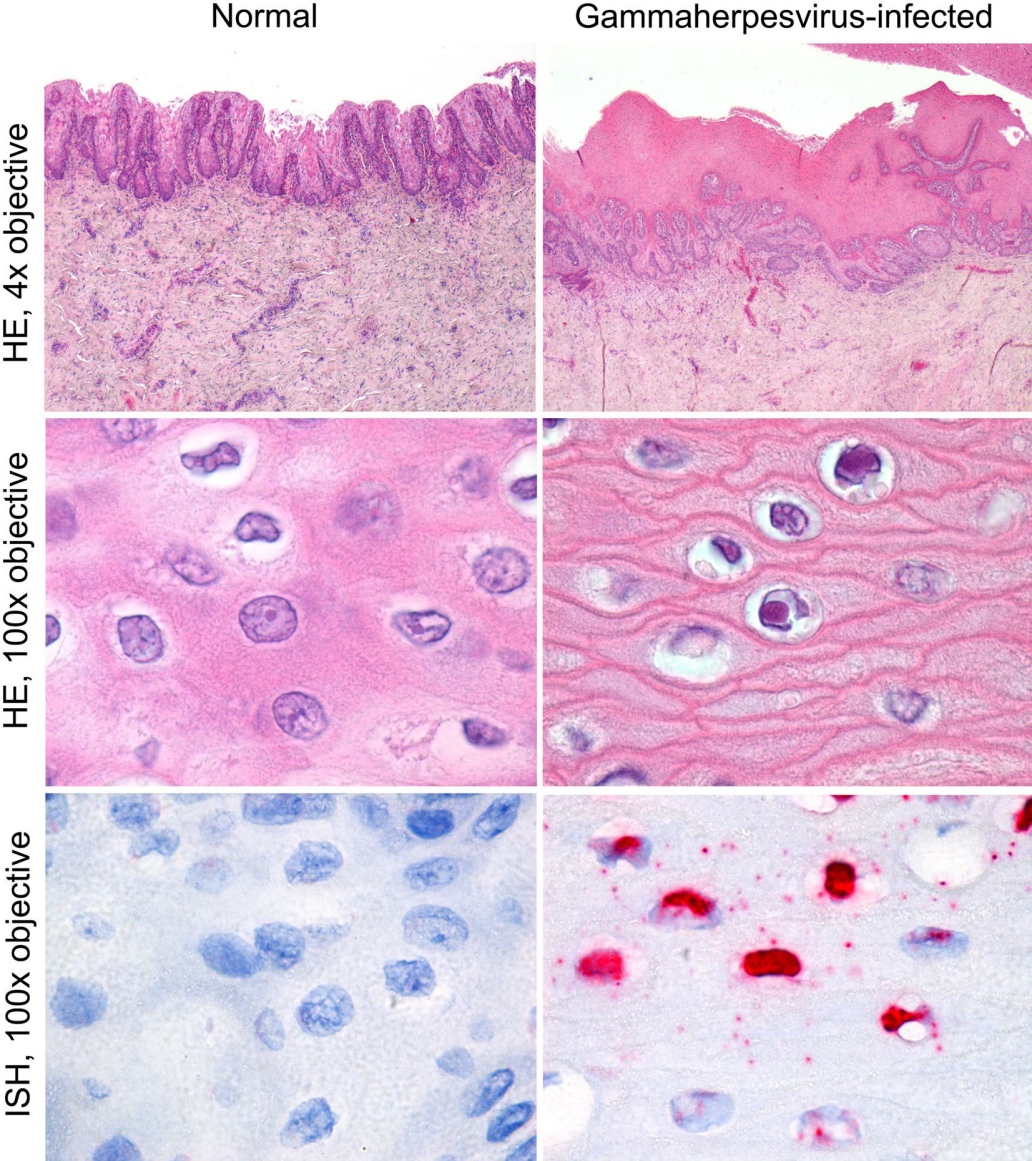
**Genital plaque associated with** ***Phocoena phocoena*** **herpesvirus type 1 (PPHV-1) infection in a harbor porpoise.** Genital mucosa of harbor porpoise #6 infected with PPHV-1, a gammaherpesvirus. Tissue sections are either stained with hematoxylin and eosin (HE) or by in situ hybridization (ISH) specific for RNA of PPHV-1. Left column shows normal genital mucosa and right column shows genital plaque infected with PPHV-1. The genital plaque is characterized by marked hyperplasia of the epithelial layer (top row). In the superficial part of this hyperplastic epithelium, many epithelial cells have nuclei with amphophilic intranuclear inclusion bodies, characteristic for herpesvirus infection (middle row). These nuclei express PPHV-2 RNA, visible as bright red staining (bottom row).

**Figure 5:**
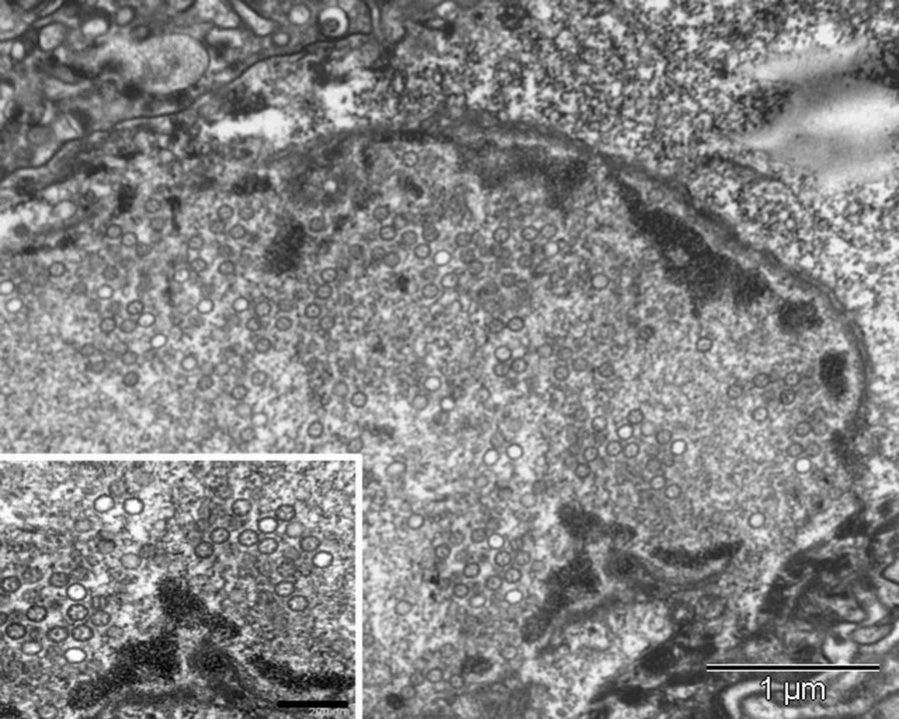
**Herpesvirus-like particles in epithelial cell of a harbor porpoise with** ***Phocoena phocoena*** **herpesvirus type 1 infection.** Genital mucosa of harbor porpoise #6 infected with *Phocoena phocoena* herpesvirus type 1, a gammaherpesvirus. Transmission electron micrographs of an epithelial cell, stained with uranyl acetate and lead citrate. The nucleus of the epithelial cell has electron-dense clumps of marginated chromatin and contains an intranuclear inclusion body. Within this inclusion body, there are round to hexagonal unenveloped viral nucleocapsids, some with an electron-dense core (inset). Bar of inset = 200 nm.

At autopsy, there was a plaque on the mucosa of the clitoris. It consisted of a round yellow-white soft nodule (18 mm long, 10 mm wide and 2 mm high) with an irregular surface. By histology, this plaque consisted of moderately thickened epithelium, characterized by hyperplasia and folding. In the upper half of the epithelium, many epithelial cells had large, round to oval, amphophilic INIB surrounded by a clear halo. The superficial connective tissue subjacent to the epithelium contained more capillaries than normal (Figure 4). By electron microscopy, the nuclei of these epithelial cells contained herpesvirus-like particles with similar characteristics as those described above, except that they had a diameter of approximately 110 nm (Figure 5). Other significant diagnoses in this porpoise were suppurative bacterial dermatitis and granulomatous pneumonia of unknown cause. By PCR, herpesvirus DNA (PPHV-1) was detected in kidney, mammary gland cyst, skeletal muscle and urinary bladder samples of this porpoise. The viral DNA polymerase 372 bp amplicon showed 100% identity with a gammaherpesvirus detected in another harbor porpoise from the Netherlands [10] (Figure 3). Other tissues from porpoise #6 (including brain, clitoral plaque, liver, lung and spleen) all tested negative for herpesvirus DNA by PCR. Virus culture from clitoral plaque, vaginal nodule, mammary gland cyst, skeletal muscle, brain, kidney, lung, spleen and urinary bladder samples of porpoise #6 was attempted, but no virus was cultured.

By next-generation sequencing of the skeletal muscle sample, which had the strongest herpesvirus DNA polymerase PCR band of all PCR-positive tissues, multiple reads of herpesvirus were obtained and used to design specific primers and obtain multiple overlapping PCR fragments. This resulted in a 1246 bp fragment homologous to gammaherpesvirus ORF56. By ISH using a probe designed based on this fragment, gammaherpesvirus RNA was detected in epithelial cells of the clitoral plaque (Figure 4). Besides herpesvirus, this skeletal muscle sample also contained four reads of papillomavirus, ranging from 232 to 330 bp and spanning a total length of 1170 bp. By BLAST analysis, the identities ranged from 73% with *Phocoena spinnipinnis* papillomavirus (accession number: AJ23837.1) for a 327 bp fragment to 83% with *Miniopterus schreibersii* papillomavirus (GenBank accession number: JQ692938.1) for a 41 bp stretch of a 232 bp fragment. The clitoral plaque sample tested negative in the papillomavirus PCR.

Based on this case report, we prospectively screened harbor porpoises that stranded alive between 2009 and 2014 (*n* = 43) for herpesvirus infection and genital plaques. These porpoises were one male neonate, 18 male juveniles, two male adults, three female neonates, 16 female juveniles and three female adults. Swabs were collected from genital slits, blowholes and oral cavities and tested for herpesvirus DNA by PCR. Of these 43 porpoises, 16 were later autopsied. Furthermore, we screened autopsy reports of the live-stranded harbor porpoises that were autopsied between 2000 and 2014 (*n* = 74). We found two additional porpoises (#7 and #8) with swabs positive for herpesvirus DNA (Table 1). In one, genital swab and blowhole swab were positive; in the other, only the blowhole swab was positive. The sequences of the PCR products from both porpoises were identical. The virus (PPHV-3) showed closest identity (86%) with cervid herpesvirus from a North American elk (*Cervus canadensis*) (Figure 3). No additional porpoises that were autopsied or that were swabbed at admission had evidence of genital plaques. Therefore, the overall prevalence of herpesvirus infection in the genital mucosa of live-stranded porpoises was 2% (1/43), while the overall prevalence of genital plaques was 1% (1/117).

## Discussion

In this study, we demonstrated infection of live-stranded harbor porpoises with three different herpesviruses: PPHV-1, previously identified by van Beurden et al. [10], and the newly discovered PPHV-2 and PPHV-3. Of these three herpesviruses, the most important as a mortality factor for harbor porpoises was PPHV-2, which was associated with severe encephalitis in a juvenile female harbor porpoise (animal #2; Table 1). Evidence that PPHV-2 infection was the cause of encephalitis were: (1) detection of characteristic INIB in neurons of the affected brain tissue by light microscopy, (2) demonstration of herpesvirus-like particles within these INIB by electron microscopy and (3) detection of PPHV-2-specific DNA in brain samples of the affected porpoise by PCR and sequencing. The combination of clinical observations, showing uncoordinated surfacing and respiration, and pathological analysis, showing severe PPHV-2-associated encephalitis, indicated that PPHV-2 infection of the brain was clinically significant and the most probable cause of stranding.

Encephalitis is commonly diagnosed in harbor porpoises, but its cause is rarely established. Encephalitis was a significant diagnosis in 9% (7/74) of porpoises in this study, 3% (4/133) of porpoises stranded along the German coast [17], and 11% (6/55) of porpoises stranded along the Belgian coast [15]. One of the Belgian encephalitis cases was caused by *Streptococcus equisimilis* sepsis and two of the German encephalitis cases were due to ß-hemolytic streptococcal septicemia. In a harbor porpoise stranded in Sweden, Kennedy et al. [12] diagnosed herpesvirus as the cause of encephalitis, based on characteristic INIB in neurons and the demonstration of herpesvirus-like particles by electron microscopy. They suspected it was an alphaherpesvirus based on immunohistochemistry. The only other cetacean species in which herpesvirus was associated with encephalitis are the bottlenose dolphin and the striped dolphin. Esperon et al. [11] detected herpesvirus DNA by PCR in the brain sample of a stranded bottlenose dolphin with a mild non-suppurative encephalitis. The DNA polymerase of the herpesvirus from the bottlenose dolphin had 98% identity with that of herpes simplex virus 1. Sierra et al. [13] detected herpesvirus DNA by PCR in the brain of a striped dolphin with severe diffuse non-suppurative meningoencephalitis. The DNA polymerase of the herpesvirus from the striped dolphin was most closely related to herpesviruses observed in the lungs of a Cuvier’s beaked whale (*Ziphius cavirostris*) and in another striped dolphin. PPHV-2, the striped dolphin herpesvirus and the bottlenose dolphin herpesvirus belong to the subfamily of *Alphaherpesvirinae*, suggesting that these viruses have a tropism for the nervous system and are potentially pathogenic in cetaceans. More extensive sampling of the brain to account for localized infection, and the application of molecular methods (e.g., PCR and next-generation sequencing) in conjunction with histological methods (e.g., immunohistochemistry and ISH) for pathogen detection are warranted to elucidate the causes of encephalitis, including herpesvirus infection, in harbor porpoises and other cetacean species.

The next herpesvirus we detected in these harbor porpoises, PPHV-1, was associated with a plaque in the clitoral mucosa of an adult female harbor porpoise (animal #6; Table 1). Evidence that PPHV-1 infection was the cause of this genital plaque were: (1) detection of characteristic INIB in epithelial cells of the affected mucosa by light microscopy, (2) demonstration of herpesvirus-like particles within these INIB by electron microscopy and (3) expression of PPHV-1-specific DNA in affected epithelial cells by ISH. The report of a similar plaque, containing herpesvirus-like particles by electron microscopy, in the penile mucosa of a harbor porpoise [28] indicates that the genital mucosa of both male and female harbor porpoises may be affected. In contrast to these genital plaques, van Beurden et al. [10] found PPHV-1 in association with skin lesions, characterized by epidermal hyperplasia and INIB.

Genital lesions associated with herpesvirus infection have been found in four other cetacean species: a male Blainville’s beaked whale (*Mesoplodon densirostris*) [29], a female Risso’s dolphin (*Grampus griseus*) [9], a male striped dolphin [30] and both male and female bottlenose dolphins [9, 31, 32]. In the Blainville’s beaked whale [29], the bottlenose dolphin [32] and the striped dolphin [30], the mucosal lesions were histologically similar to those reported in harbor porpoise #6: a well-demarcated area of epithelial hyperplasia, characterized by INIB in affected epithelial cells. In these four cetacean species, as well as in the harbor porpoise, the herpesvirus associated with these genital lesions belonged to the subfamily *Gammaherpesvirinae*. In a zoo collection of bottlenose dolphins, infection was endemic and seroconversion occurred around the age of onset of sexual behavior [32]. This epidemiological observation, together with the predilection for the genital mucosa, indicates that sexual contact is an important route of transmission of gammaherpesviruses in cetaceans.

There is ongoing debate about the roles of herpesvirus infection and papillomavirus infection as the cause of genital lesions in cetaceans [33]. In that perspective, it is of interest that papillomavirus was detected in a skeletal muscle sample of harbor porpoise #6 by next-generation sequencing. Although we did not detect papillomavirus by PCR in the clitoral plaque sample of this animal, it cannot be excluded that the primers used—based on papillomaviruses from Burmeister’s porpoise—are not suited to detection of papillomavirus of harbor porpoises. Therefore, the co-involvement of papillomavirus infection in the etiology of genital lesions in harbor porpoises needs to be explored further.

The last herpesvirus we detected in harbor porpoises, PPHV-3, was not related to disease. Despite detection in brain, pulmonary lymph node, genital slit and blowhole samples of several porpoises (animals #1, #3, #4, #5, #7 and #8; Table 1), no association with pathologic changes in the respective tissues was observed. It remains to be determined why PPHV-3 had an apparent predilection for juvenile male harbor porpoises and what the pathogenic potential of this virus is.

Because this research was part of a long-term study of morbidity and mortality factors in live-stranded harbor porpoises, we had a substantial sample size to evaluate the prevalence of both herpesvirus-associated encephalitis and genital plaques. The prevalence of PPHV-2-associated encephalitis was low (1%; 1/74), indicating that this morbidity factor is rare in harbor porpoises that strand alive on the Dutch and adjacent coasts. However, a caveat is that only one brain sample (a sample of cerebral tissue and a sample of cerebellar tissue in one vial) was collected for virological analysis as part of our standard autopsy protocol. Given that herpesvirus infections may be limited to certain areas of the brain [34], we may have underestimated the number of porpoises with PPHV-2 in the brain.

The low prevalence of herpesvirus-associated genital plaques in live-stranded harbor porpoises—0% (0/52) in juvenile and adult males, 2% (1/46) in juvenile and adult females—contrasts with the much higher prevalence in bottlenose dolphins in a zoo collection—23% (3/13) in juvenile and adult males, 27% (4/15) in juvenile and adult females [32]. Similarly, regardless of the presence or absence of genital plaques, PPHV-1 infection was undetectable in the genital slit of harbor porpoises—0/20 for juvenile and adult males, 0/19 for juvenile and adult females—compared to a much higher prevalence of *Tursiops truncatus* herpesvirus type 1 infection in bottlenose dolphins in a zoo collection—19% (4/21) for juvenile and adult males, 33% (5/15) for juvenile and adult females [32]. Possible explanations are differences in herpesvirus, host species, or captive versus free-living populations.

To obtain a better idea of the prevalence of infection with these herpesviruses in autopsied harbor porpoises, it would be important to determine the site of latent infection and to sample those sites. For example, herpes simplex virus 1 in human beings establishes a latent infection in the root ganglia of the trigeminal nerve [35]. The development of serological assays to detect specific antibodies against PPHV-1, PPHV-2, and PPHV-3 would make it possible to estimate the prevalence of infection in live harbor porpoises. Such a serological assay, based on cultured virus, was successfully developed to detect specific antibodies against *Tursiops truncatus* herpesvirus in bottlenose dolphin sera [32].
